# Vessel architecture imaging reveals microvascular rarefaction and capillary-to-arteriole shift in cerebral small vessel disease

**DOI:** 10.1177/0271678X251358968

**Published:** 2025-07-31

**Authors:** Paulien HM Voorter, Maud van Dinther, Gerhard S Drenthen, Elles P Elschot, Julie Staals, Robert J van Oostenbrugge, Jacobus FA Jansen, Walter H Backes, on behalf of the CRUCIAL consortium

**Affiliations:** 1 Department of Radiology and Nuclear Medicine, Maastricht University Medical Center, Maastricht, the Netherlands; 2 Mental Health and Neuroscience Research Institute, 5211 Maastricht University, Maastricht, the Netherlands; 3 Cardiovascular Disease Research Institute, 5211 Maastricht University, Maastricht, the Netherlands; 4 Department of Neurology, Maastricht University Medical Center, Maastricht, the Netherlands; 5 Department of Electrical Engineering, 3169 Eindhoven University of Technology, Eindhoven, the Netherlands

**Keywords:** Capillary reduction, perfusion MRI, vascular morphology, vessel size imaging, white matter lesions

## Abstract

In cerebral small vessel disease, clinical imaging markers such as white matter hyperintensities are often considered late-stage indicators, with limited understanding of (early) underlying microvascular alterations. This prospective, cross-sectional study utilized vessel architecture imaging to assess microvascular alterations *in vivo* in 40 patients with cerebral small vessel disease and 21 controls at 3T MRI. Quantitative measures for vessel density, radius, and vessel type composition were derived. Overall, patients had lower vessel density and larger microvessel radius than controls, while blood perfusion levels remained similar between groups. Moreover, a shift from capillaries to arterioles was observed in cortical gray matter and normal-appearing white matter in patients. White matter hyperintensities demonstrated lower vessel density and larger radii than normal-appearing white matter, with no difference in vessel type composition. Our findings support the role of microvascular rarefaction in the pathophysiology of cerebral small vessel disease. The remaining vascular network with relatively more larger or more dilated (arteriolar) blood vessels and less dense microvessels may reflect uneven flow patterns, leading to less efficient delivery of oxygen, nutrients, and removal of metabolic waste products. Vessel architecture imaging can provide an early biomarker in future trials on microvascular growth therapy.

**Clinical Trial Registration Information:** Measuring the blood vessel density in patients with heart failure or reduced cognitive function of vascular origin: CRUCIAL. https://doi.org/10.1186/ISRCTN22301128. 848109

## Introduction

Cerebral small vessel disease (cSVD) is a group of disorders of the cerebral arterioles, capillaries, and venules. The most prevalent type is the age- and cardiovascular risk factor associated deep perforator arteriopathy, further called cSVD.^1^ cSVD is the main cause of vascular cognitive impairment. Furthermore, it may cause lacunar stroke, and these patients are also at increased risk for vascular cognitive impairment.^2^ Animal models and post-mortem studies in humans point to a role for microvascular rarefaction in the pathophysiology of several small vessel disorders, including small vessel disease in the brain.^3–6^ Microvascular rarefaction is the functional reduction in perfused microvessels and/or structural reduction in microvascular density. Microvascular rarefaction can result in chronic hypoxia and shortage of nutrients, and may in turn lead to neural dysfunction and brain tissue damage.^3,7^

Conventional MRI cannot visualize the smallest cerebral blood vessels morphologically in humans *in vivo*, due to a limited spatial resolution. Alternatively, cerebral vessel architecture imaging (VAI), an advanced MRI technique that involves integrated hybrid spin-echo (SE) and gradient-echo (GE) perfusion scans, can provide information to characterize the microvascular morphology, as SE perfusion is selectively sensitive to microvessels (radius <10 μm) and GE perfusion to all vessel sizes.^8–11^ As such, VAI can provide quantitative information about the vessel density (Q), radius (vessel size index; VSI), and the composition of vessel types (microvessel type indicator; MTI) in a voxel.^8,9,12^

Using VAI, we aim to investigate abnormalities of the microvascular bed in patients with cSVD compared to controls. We hypothesize that patients with cSVD exhibit microvascular rarefaction, and thus have lower vessel density than controls, possibly compensated by larger calibers of the remaining vessels.

## Material and methods

### Study population

We prospectively recruited patients for this research study with clinically overt cSVD, comprising patients with vascular cognitive impairment or lacunar stroke, and age- and sex-matched normocognitive controls between October 2021 and October 2023 at the Maastricht University Medical Centre, the Netherlands. Participants with vascular cognitive impairment or lacunar stroke were recruited from the memory clinic or stroke clinic. Vascular cognitive impairment due to cSVD was defined as the presence of all three criteria: (1) subjective complaints of cognitive functioning, (2) objective cognitive impairment in at least one cognitive domain in neuropsychological assessment or a Montreal Cognitive Assessment (MoCA) score <26, and (3) imaging evidence of cSVD,^13^ defined as extensive leukoaraiosis on CT, or moderate to severe WMH on MRI (Fazekas score ≥2), or mild WMH (Fazekas score = 1) in combination with lacunes or microbleeds.^14^ Severe cognitive impairment defined as a Clinical Dementia Rating score >1 was an exclusion criterion. Lacunar stroke due to cSVD was defined as a clinical lacunar stroke syndrome with a compatible lesion on CT or MRI, and additional imaging evidence of cSVD (mild to severe WMH and/or microbleeds). Lacunar stroke patients were included at least three months post-stroke to avoid acute stroke effects.

Age- and sex-matched controls were recruited from the outpatient clinic of Neurology, where they presented with complaints related to the peripheral nervous system, such as mononeuropathy, chronic polyneuropathy, or backpain. These diagnoses are not expected to have effect on the brain microvasculature. Controls had no overt cerebrovascular diseases, and no cognitive impairment (Mini-Mental State Examination (MMSE) > 24).

General exclusion criteria for both patients with cSVD and controls included the presence of other neurological or psychiatric conditions affecting the brain (including multiple sclerosis, Parkinson’s disease, major cortical stroke, major neuro-trauma, and brain tumours), and contraindications for MRI or gadolinium contrast agent.

The study has been approved by the Medical Ethics Committee of Maastricht University Medical Centre. Written informed consent was obtained from all participants according to the Declaration of Helsinki. This study adhered to the applicable items in the STROBE checklist and was part of the CRUCIAL study, a multicentre research program on the role of microvascular rarefaction in vascular dementia and heart failure (Trial Registration: ISRCTN22301128).^15^

### MRI acquisition

All study participants underwent whole-brain imaging on a 3 Tesla MRI system (Ingenia CX, Philips Healthcare, Best, the Netherlands) using a 32-element head coil. The imaging protocol included structural imaging, VAI, and diffusion-weighted imaging.

Structural images comprised T_1_-weighted images with two different inversion times and T_2_-weighted fluid-attenuated inversion recovery (FLAIR) scans for anatomical reference and visualization of white matter hyperintensities (WMH), see Table S1 for details on scan parameters.

For VAI, we used a dynamic perfusion protocol with a rapid transverse single-shot combined GE SE echo-planar-imaging sequence (Gyrotools LLC, Zurich, Switzerland) using the following acquisition settings: TR = 1634 ms, TE_GE_/TE_SE_ = 25/78 ms, FA = 90°, matrix size = 76 × 61, acquired in-plane resolution = 3.0 mm × 3.3 mm, number of slices = 16, slice thickness = 5 mm, slice gap = 2 mm, number of dynamics = 100, dynamic scan interval = 1.6 s, total acquisition time = 2:50 min:s, SENSE factor = 2. Contrast agent (0.1 mmol gadobutrol (Gadavist®) per kg body weight) was injected intravenously with an injection rate of 3 mL/s at the beginning of the 9^th^ dynamic acquisition volume, followed by a 20 mL saline flush. One GE and one SE volume were acquired before the dynamic series with the opposite phase-encoding direction to allow for geometric echo-planar-imaging distortion correction. Approximately 30 minutes before the VAI acquisition, a contrast preload (0.1 mmol gadobutrol (Gadavist®) per kg body weight) was administered to the subject, which would significantly reduce any intravascular T_1_-weighted signal enhancing and possible extravascular T_1_ effects of contrast agent leakage during the VAI scans.^16^

To calculate the diffusion coefficient (*D*) of the brain parenchyma, diffusion-weighted MR imaging was performed using a single shot spin-echo echo-planar-imaging inversion-recovery sequence (TR/TE/TI = 6800/86/2230 ms). The diffusion gradients were applied in three orthogonal directions with b-values of 0, 200, 300, 400, 500, 600, 800, and 1000 s/mm^2^. To correct for echo-planar-imaging distortions, we additionally acquired the image without diffusion-weighting (b = 0 s/mm^2^) in the opposite phase-encoding direction.

## MRI analysis

### Brain segmentation

The regions of interest (ROIs), that is deep gray matter (DGM), cortical gray matter (CGM), normal-appearing white matter (NAWM), and WMHs were automatically segmented from the T_2_-FLAIR and T_1_-weighted images using automated image analysis software (*samseg*^17^) followed by visual inspection and manual corrections (P.V.). Because the periventricular white matter is often affected in patients, while often normal in controls, this leads to spatial differences in the NAWM between patients and controls (more periventricular white matter included in NAWM ROI in controls). To minimize such location-dependent effects of NAWM, we excluded a zone of 1 cm around the ventricles. Furthermore, any infarcts, hemorrhages, developmental venous anomalies, and cavernomas were manually segmented (M.v.D.) and excluded from the ROIs. All manual corrections and segmentations were performed under the supervision of an experienced vascular neurologist (J.S.). The resulting ROIs were transformed to the vessel architecture image space (*FSL flirt*^18^).

### Vessel architecture imaging analysis

The dynamic GE and SE images were corrected for head motion (*FSL mcflirt*^18^) and geometric distortions (*FSL topup*^19^). Hereafter, the dynamic change in *R*_2_* and *R_2_* relaxation rate time were calculated (from GE and SE, respectively) per ROI by dividing their signals by the average precontrast signals *S*_0_: Δ*R*2*(*t*) = −*ln*(*S*_*GE*_(*t*)/*S*_*GE*_,_0_)/*TE*_*GE*_ and *ΔR*_2_(*t*) = −*ln(S_SE_(t)/S_SE,0_)/TE_SE_*. The vessel density index (Q) (unit: s^−1/3^) was derived by *Q = ΔR*_2_/(Δ*R*_2_*)^2/3^ and using only the change in relaxational rates during the first-pass bolus (4 timepoints (6.4 s) before and 8 timepoints (12.8 s) after the bolus peak) to exclude the data points with no or too little changes in relaxation rates.^20^

Furthermore, a quantitative measure for the weighted average of blood vessel radii (VSI, unit: μm) was obtained according to^20^: *VSI* = 0.867·*(D*·*rCBV)^1/2^*·*Q*^−3/2^.^20^ Here, *D* is the parenchymal diffusion coefficient, derived from the geometric-distortion-corrected diffusion images. A mono-exponential function was fitted to the diffusion signal decay for b ≥200 s/mm^2^ to obtain *D*.^21^ The rCBV is the relative cerebral blood volume, derived from the dynamic GE images as described previously.^22^ We semi-automatically selected voxels in the middle and posterior cerebral arteries to derive the arterial input function (AIF) and fitted a gamma-variate function to both the AIF and the tissue concentration time course derived from the GE sequence, *C(t)* ∝  ΔR2*(*t*).^23,24^ The AIF was scaled in such a way that the obtained rCBV in NAWM was set to 3.2% using *rCBV* = $\int_{0}^{\infty }C\left(t\right)dt/\int_{0}^{\infty }\mathrm{AIF}\left(t\right)dt$ , similar to.^22^ We excluded voxels with the 10% highest rCBV values from our analyses to avoid signal contamination from the largest blood vessels.^23^ Furthermore, the cerebral blood flow (CBF) was separately calculated from the GE (perfusion through all vessels) and SE (perfusion through small vessels [radius < 10 μm]) sequence using a block-circulant singular value decomposition method.^23,25^

**Figure 1.**
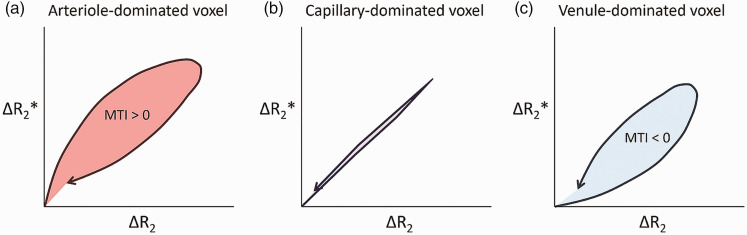
Conceptual illustration of the vortex curves. Vortex curves for an arteriole-dominated voxel (a), a capillary-dominated voxel (b), and a venule-dominated voxel (c). For an arteriole-dominated region, the contrast agent bolus has arrived in the arterioles, causing a significant susceptibility-induced effect, but not yet in the capillaries or venules, and only the larger arterioles contribute to the gradient echo signal changes (Δ R_2_*), while the capillaries contribute only later to the spin echo signal changes (Δ R_2_), explaining the clockwise loop over time. For a venule-dominated region, the capillaries are already filled with gadolinium contrast agent and contribute to the spin echo signal changes (Δ R_2_), before the venules become filled and start contributing to the gradient echo signal changes (Δ R_2_*), explaining the counter-clockwise loop over time.

The *R*_2_*(*t*) was plotted against the *R*_2_(*t*) to generate the vessel vortex curve.^12^ The area within this vortex curve loop (i.e., mean vessel type indicator [MTI]) was calculated (positive for a clockwise loop and negative for a counter-clockwise loop transversed clockwise) (Figure 1).^26^ The MTI is dependent on blood oxygenation levels and the vessel radius.^12,27^ Since these characteristics differ among arterioles, capillaries, and venules, the MTI can offer insights into the proportion of these vessel types within a voxel.^12^ A positive MTI value indicates a higher presence of arterioles, while a negative MTI value suggests a greater presence of venules.^12^ Additionally, the cerebral blood flow (CBF) was separately calculated from the GE (perfusion through all vessels) and SE (perfusion through small vessels [radius <10 μm]).^10^

**Figure 2.**
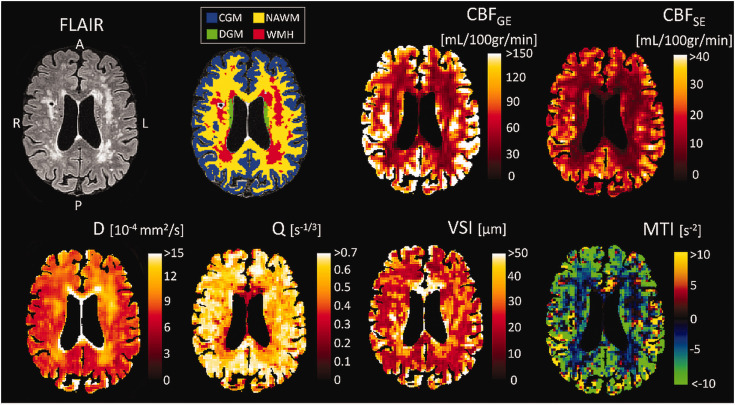
Examples of the MRI-derived images. Example of a FLAIR image, brain segmentation, cerebral blood flow derived from gradient echo (CBF_GE_), cerebral blood flow derived from spin echo (CBF_SE_), diffusion coefficient (D), vessel density index (Q), vessel size index (VSI), and microvessel type indicator (MTI) image for a cSVD patient. Both CBF maps depict higher perfusion in gray matter compared to white matter and hypoperfusion in white matter hyperintensities (WMH). D is highest in WMH, indicating microstructural damage. Q is lower and VSI is higher in WMH compared to surrounding white matter. Overall, the brain tissue has a negative MTI value, indicating a larger volume fraction of the venules/veins compared to arterioles/arteries. CGM: cortical gray matter; NAWM: normal-appearing white matter; DGM: deep gray matter.

We also performed the analysis described above on a voxel-wise basis to observe the quality and spatial pattern of the calculated MR parameters (Figure 2). We used the VSI image to investigate the distribution of the vessel radii within the ROIs. The distribution of the VSI was visualized using a histogram and quantified by the interquartile range (IQR) in NAWM, DGM, and CGM.

### General and health characteristics

Cardiovascular risk factors, i.e. hypertension, diabetes mellitus, hypercholesterolemia, body mass index, smoking, and alcohol use, were obtained from self-reported medical history and medication use. Excessive alcohol use was determined as >21 units per week for males and >14 units per week for females.

### Statistical analysis

Characteristics of the study population were presented as mean ± standard deviation (SD), as median [IQR], or as number (percentages). Independent samples t-test and Pearson X^2^ were performed to compare general and health characteristics between patients with cSVD and controls.

VAI measures (Q, VSI, and MTI as dependent variables) in NAWM, DGM, and CGM, were compared between patients and controls (group as independent variable) using univariable linear regression analyses. Additionally, multivariable linear regression analyses accounting for age and sex (covariables) were performed. Here, a total sample size of 60 is needed to detect a moderate effect size d of 0.6–0.7 (G*Power; difference between two independent means, beta = 0.2 and alpha = 0.05).

As a previous study showed a significant decrease in vessel density with age in controls but not in cSVD patients,^4^ we assessed effect modification by including an interaction term (patient group × age). In an additional exploratory analysis, we added one cardiovascular risk factor at a time (hypertension, diabetes mellitus, hypercholesterolemia, smoking, excessive alcohol use, or body mass index) as an independent variable to the multivariable regression model that included patient group, sex, and age to test whether adding these cardiovascular risk factors influenced the interpretation of the main results.

Furthermore, differences in VAI measures (Q, VSI, and MTI) between NAWM and WMH were tested by paired t-tests within the patient group, as only patients had substantial WMH volumes.

Lastly, we performed independent samples t-tests to examine the difference in the voxel-wise VSI distribution (quantified by a subject-specific IQR) within DGM, NAWM, and CGM between patients and controls.

All analyses were performed with SPSS software (v28.0; IBM, Chicago). A *P*-value less than 0.05 was considered statistically significant.

## Results

For this study, 40 patients with cSVD (lacunar stroke *n = *11, vascular cognitive impairment *n = *29) and 21 controls were included. Table 1 summarizes the study population’s characteristics. Patients more often had a history of hypercholesterolemia and stroke, had a lower MMSE score, and had a larger WMH volume compared to controls.

**Table 1.** Characteristics of the study population.

Characteristic	Patients with cSVD(*n* = 40)	Controls(*n = *21)	*P*-value
Age [years]	70.1 ± 9.3	67.0 ± 6.5	0.131
Sex [n, (% women)]	13 (32.5)	6 (28.6)	0.753
Hypertension [n, (%)]	32 (80.0)	13 (61.9)	0.127
Diabetes mellitus [n, (%)]	6 (15.0)	3 (14.3)	0.629
Hypercholesterolemia [n, (%)]	36 (90.0)	12 (57.1)	**0.006**
Smoking, currently [n, (%)]	6 (15.0)	3 (14.3)	1.000
Excessive alcohol use [n, (%)]	2 (5.0)	0 (0.0)	0.541
BMI [kg/m^2^]	27.3 ± 4.1	26.2 ± 3.3	0.291
History of stroke [n, (%)]	24 (60.0)	0 (0.0)	**<0.001**
History of TIA [n, (%)]	14 (35.0)	3 (14.3)	0.086
MMSE	26.8 ± 3.8	28.9 ± 1.3	**0.003**
WMH volume [cm^3^]	20.2 [8.4–39.0]	3.1 [1.1–6.1]	**<0.001**

Data are presented as means ± standard deviation, or median [interquartile range], or number of participants (percentages).

cSVD: cerebral small vessel disease; BMI: body mass index; TIA: transient ischemic attack; MMSE: Mini-Mental State Examination; WMH: white matter hyperintensity.

**Figure 3.**
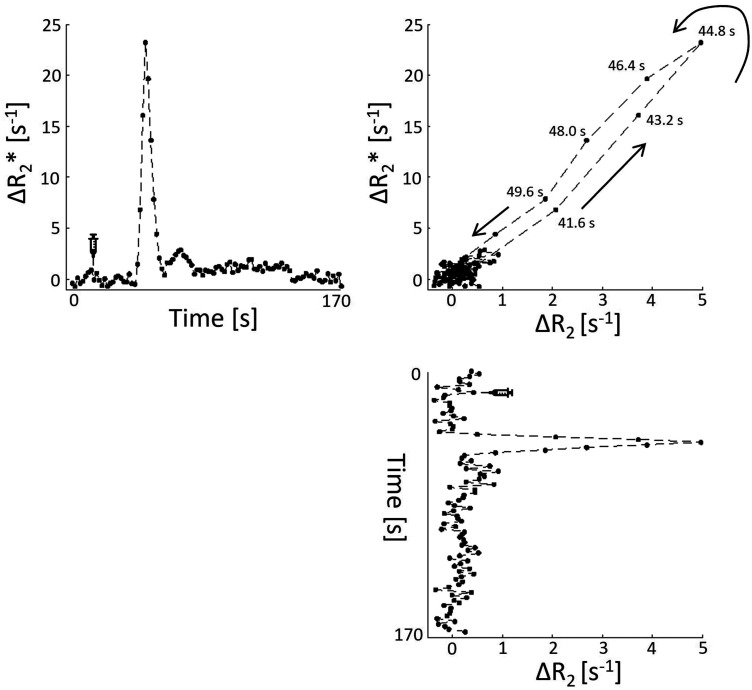
*In vivo* example of changes in relaxation rates. A vessel vortex curve (top right) is derived from the gradient echo signal changes (Δ R_2_*) over time (top left) and the spin echo signal changes (Δ R_2_) over time (bottom right) for a representative voxel in the deep gray matter of a patient with cSVD.

Figure 3 shows an example of the changes in relaxation rates over time and a vessel vortex curve for a deep gray matter voxel of a patient with cSVD.

### VAI parameters between patients and controls

**Figure 4.**
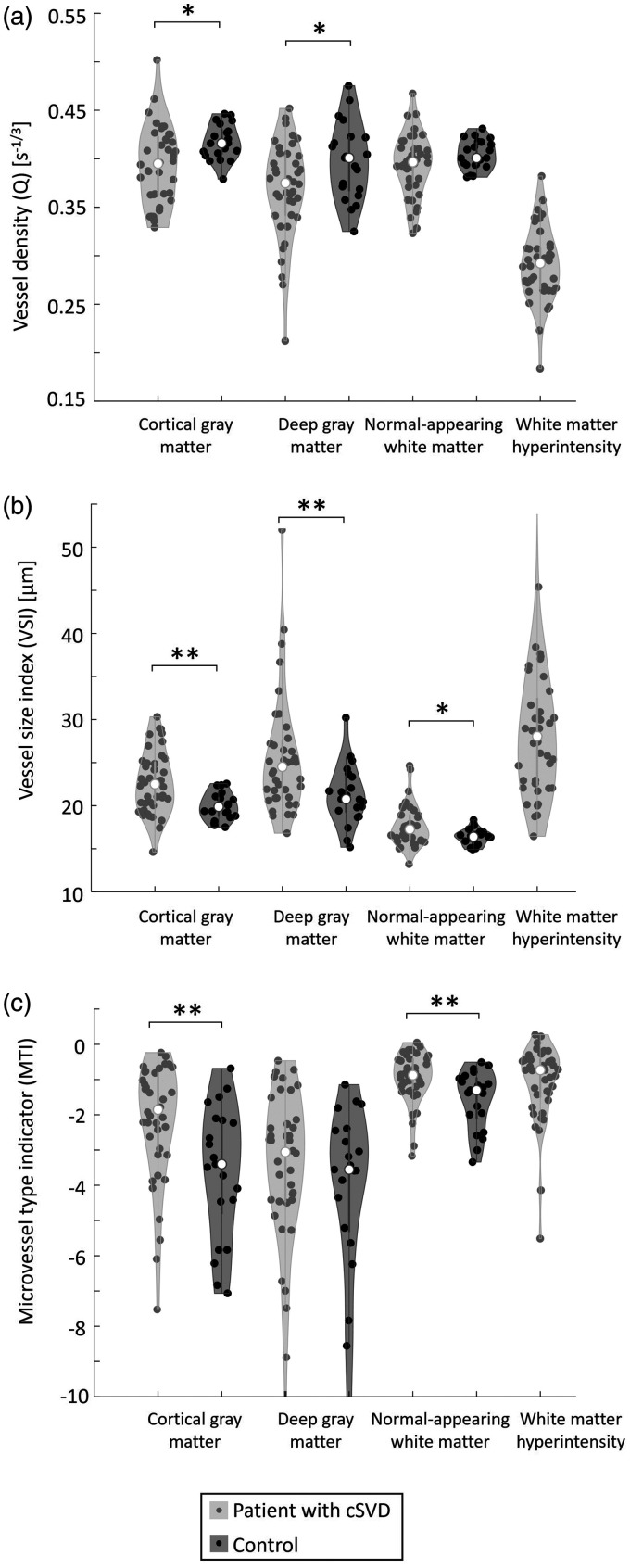
Vessel architecture imaging measures in several brain regions for patients with cSVD and controls. (a) vessel density, (b) vessel size index, and (c) microvessel type indicator. *P < 0.05, **P < 0.01, only indicated for group comparisons. cSVD: cerebral small vessel disease.

Figure 4 presents an overview of the VAI measures in patients and controls (numerical values are also presented in Table S2). Q was lower in patients than in controls for DGM and CGM, but not for NAWM. A higher VSI was found in patients compared to controls for all ROIs. MTI was negative for both patients and controls in all ROIs. In NAWM and CGM, MTI was significantly less negative (i.e., higher) in patients compared to controls. These results remained significant after controlling for age and sex (Table 2), and also after additional correction for various cardiovascular risk factors (Table S3). There were no significant differences in CBF, either SE- or GE-derived, between cSVD patients and controls (Table S2).

**Table 2.** Regression coefficients of multivariable linear regression analyses for vessel architecture measures.

	Covariable group(0 = control, 1 = patient with cSVD)		Covariable age (in years)		Covariable sex(0 = female, 1 = male)	
	β (95% CI)	*P*-value	β (95% CI)	*P*-value	β (95% CI)	*P*-value
*Q*						
NAWM	−0.009 (−0.022–0.004)	0.177	−0.001 (−0.002–0.000)	**0.045**	−0.027 (−0.041– −0.014)	**<0.001**
DGM	−0.026 (−0.049– −0.003)	**0.028**	−0.002 (−0.003–0.000)	**0.023**	−0.041 (−0.064– −0.018)	**<0.001**
CGM	−0.019 (−0.033–−0.006)	**0.007**	−0.002 (−0.003– −0.001)	**<0.001**	−0.039 (−0.053−0.025)	**<0.001**
*VSI*						
NAWM	1.144 (0.189–2.098)	**0.020**	0.079 (0.025–0.133)	**0.005**	1.722 (0.750–2.694)	**<0.001**
DGM	3.994 (1.016–6.972)	**0.009**	0.213 (0.045–0.382)	**0.014**	4.624 (1.592–7.656)	**0.003**
CGM	2.339 (1.010–3.668)	**<0.001**	0.177 (0.102–0.252)	**<0.001**	1.646 (0.787–2.504)	**<0.001**
*MTI*						
NAWM	0.572 (0.193–0.951)	**0.004**	0.023 (0.002–0.045)	**0.036**	0.655 (0.269–1.040)	**0.001**
DGM	0.038 (−1.146–1.221)	0.949	0.129 (0.062–0.196)	**<0.001**	2.799 (1.594–4.004)	**<0.001**
CGM	1.256 (0.413–2.099)	**0.004**	0.066 (0.018–0.114)	**0.008**	1.646 (0.787–2.504)	**<0.001**

The bold values denotes are significant (*P* < 0.05). cSVD: cerebral small vessel disease; CI: confidence interval; Q: microvessel density: VSI: vessel size index; MTI: microvessel type indicator; NAWM: normal-appearing white matter; DGM: deep gray matter; CGM: cortical gray matter.

Noteworthy, older age and male sex were associated with lower Q, higher VSI, and higher MTI for all ROIs (Table 2 and Figure S1). There was no significant interaction between patient group and age for Q, VSI, or MTI (results not shown). Associations between cardiovascular risk factors and VAI measures can be found in Table S3.

### VAI parameters between NAWM and WMH in patients

Q was 26% lower in WMH compared to NAWM (0.29 ± 0.04 versus 0.39 ± 0.03 s^−1/3^, *P < *0.001), whereas VSI was 64% higher in WMH compared to NAWM (29.0 ± 9.7 versus 17.7 ± 2.3 μm, *P < *0.001) in the cSVD patients (Table S2). MTI did not differ between WMH and NAWM (−1.1 ± 1.1 versus −0.9 ± 0.7, *P = *0.35).

### Voxel-wise vessel radius distribution

**Figure 5.**
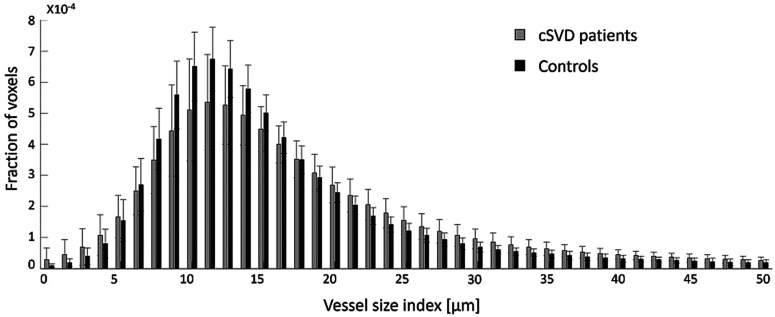
Vessel size index distribution in deep gray matter. The distribution of the vessel size index is wider in cSVD patients compared to controls, reflecting a more inhomogeneous microvascular bed in cSVD patients. Note that the fraction of voxels containing a vessel size index above 50 μm is small and not included in this figure. cSVD: cerebral small vessel disease.

Patients with cSVD had a wider voxel-wise VSI distribution compared to controls in NAWM (IQR(VSI) = 9.1 μm versus IQR(VSI) = 7.9 μm, *P = *0.004), in DGM (IQR(VSI) = 13.6 μm versus IQR(VSI) = 10.8 μm, *P = *0.003), and in CGM (IQR(VSI) = 15.1 μm versus IQR(VSI) = 12.6 μm, *P = *0.002). Figure 5 visualizes the VSI distribution in DGM for patients and controls (see Figure S2 for the VSI distribution in CGM and NAWM).

## Discussion

In the current study, we assessed the cerebral microvascular morphology of patients with cSVD and controls using VAI. Patients with cSVD were found to have a lower vessel density, larger vessel size, and a more arteriole-dominated vessel type, compared to sex- and age-matched controls, under nearly normal global cerebral perfusion conditions. Similar differences were found when we compared the microvasculature in WMH to the NAWM in patients with cSVD. Moreover, we showed that these alterations in the microvascular architecture are associated with higher age and male sex.

## Microvascular architecture in cSVD

The larger vessel radius found in cSVD and in ageing, could imply that blood vessels dilate to compensate for local hypoperfusion at the microscale, and/or that the smallest capillaries collapse or disappear (capillary rarefaction), increasing the weighted-average VSI due to more contribution of the relatively larger blood vessels. The increased MTI in cSVD and ageing points towards a shift from capillary to arteriolar vessel types and/or regression of venules,^12,28^ whereas the decreased Q is in accordance with the disappearance of the (smallest) vessels. As such, our findings support the role of microvascular rarefaction in the pathophysiology of cSVD. Moreover, the observed differences between patients and controls in terms of vessel density, vessel size, and composition of vessel types are in line with the concept of cSVD as a whole-brain disease, with widespread microvascular change that is not limited to white matter and deep gray matter, but also involves the cortex.^29^ We did not find a significant difference between patients and controls in (neither GE nor SE derived) CBF. This approximately equal level of cerebral perfusion might be explained by the balancing combination of decreased vessel density and increased vessel radii. As such, VAI can be regarded as a powerful technique sensitive to early (functional) microvascular changes in cSVD that would occur before resting CBF reduces.^30^

The microvascular architecture metrics derived in our study are within the ranges found in previous literature,^28,31,32^ but *in vivo* studies on microvessel architecture in cSVD are sparse. A single small previous study by Choi *et al.*^32^ applied vessel size imaging in 10 patients with vascular dementia and 12 elderly controls. Although this study applied a different vessel size imaging method, which involves R2* and R2 mapping before and after contrast administration (i.e., steady-state instead of our dynamic sequence),^32^ their results align well with our finding of an increased VSI in cSVD patients compared to controls.

Our results are mostly in line with human post-mortem pathology studies, which demonstrated a decreased microvascular density in WMH compared to NAWM, and in patients with cSVD compared to elderly controls, both inside and outside the lesions.^4,5,33^ However, although we indeed showed differences in microvessel density in DGM and CGM between patients with cSVD and controls, we were not able to confirm these differences in the NAWM. It is very plausible that WMH develop at places that have a relatively low vascular density by origin, which may lead to a paradoxical effect of losing low-density areas in the NAWM in the patient group. Another explanation could be the relatively high age of our study population (39% over 70 years of age). The substantial age-related decline in vascular density in the brains of elderly might have overshadowed a disease effect.^4,33^ In line with our results, Choi *et al.*^32^ (mean age 78 years in patients and 74 years in controls) did also not demonstrate a difference in vessel density in NAWM between patients and controls.

Postmortem studies assessing vessel size and dominating vessel type in cSVD are sparse. One post-mortem study^5^ demonstrated significantly wider capillaries in the white matter, but not the gray matter, in patients with vascular dementia (*n = *13) compared to ageing controls (*n = *16). Likewise, we showed a larger vessel radius in cSVD patients compared to controls, although we demonstrated differences in both white matter and gray matter. Nonetheless, our VSI measure comprises a wider variation of small vessel types (up to 50 μm), whereas the previous study^5^ only assessed capillaries (up to 10 μm).

Moreover, we investigated changes in vessel radius in more depth by looking at its voxel-wise distribution per brain region. Overall, the cSVD patients had a wider vessel radius distribution than controls, suggesting both microvessel constriction and dilation. This could be associated with enhanced pericyte activity (microvessel constriction) and pericyte loss (microvessel dilation), resulting in disrupted and uneven flow patterns within the vascular network.^6^

The alterations in microvasculature architecture that were observed in cSVD can have detrimental effects on the brain tissue. Exchange of oxygen, nutrients, and metabolic waste products occurs in capillaries. Both a shift from a capillary to an arteriolar vessel type and a decrease in microvessel density result in a decreased capillary surface area available for gas and molecule exchange. Moreover, the uneven flow patterns in the vascular network, which are reflected by the wider vessel size distribution in cSVD patients, can result in increased variations in capillary transit time (heterogeneity) and thereby inefficient delivery of oxygen and nutrients to the tissue in the remaining perfused capillaries (functional shunting).^34^ Chronic shortage of oxygen and nutrients in brain tissue can consequently give rise to microstructural tissue damage and eventually contribute to macrostructural lesions visible on MRI.^3,7^

## Microvascular architecture in relation to age and sex

We demonstrated an association between older age and lower vessel density, larger vessel size, and a shift from capillary to arteriolar vessel types. Our findings are all in line with the results of the contralateral hemisphere in a previous VAI study with unifocal low-grade glioma patients.^28^ The larger vessel radius with increasing age also matches the finding of capillary and arteriolar dilatation with ageing that has been demonstrated in human post-mortem studies and in animal models.^35–37^ Our observation of an association between increasing age and a shift from capillaries to an arteriole-dominated profile (i.e., higher MTI), is also in accordance with a previous post-mortem study that showed an increased percentage of arterioles with ageing in patients without clinical or pathological evidence of central nervous system disorders (*n = *5 with a mean age of 38 years and *n = *5 with a mean age of 74 years).^35^ A decline in vessel density with age has also been confirmed by human post-mortem studies, although it was shown that the rate of the age-associated decrease in vascular density differed between patients with cSVD and controls.^4,33^ In controls, the vessel density significantly decreased with age between 55 and 90 years, whereas in patients with cSVD, vessel density was already substantially reduced at the ages of 55–60 years and did not significantly decrease further.^4,33^ In our study with a mean age of almost 70 years, we did not find an interaction between patient group and age, suggesting comparable effects of age on vessel density in both cSVD patients and controls.

Furthermore, our study unveils distinct sex-specific patterns in cerebral microvascular architecture. We demonstrated a lower vessel density, larger vessel size, and more arterioles or fewer venules (i.e., higher MTI) in males compared to females. These findings fit the higher prevalence of severe cSVD found in males compared to females.^38^ Moreover, the aforementioned VAI study in the contralateral hemisphere of low-grade glioma patients reported similar findings of sex-specific differences in vessel architecture.^28^ However, they mainly recruited premenopausal women,^28^ while in our study, the vast majority of women were postmenopausal. Next to biological factors, other factors that could contribute to the observed sex-specific differences include variations in risk factor exposure, adherence to risk factor interventions, or sample bias as we included substantially more males (69%) than females (31%).

## Clinical perspective

The results of our study offer a more detailed understanding of microvascular alterations in cerebral small vessel disease compared to blood perfusion only. VAI may sense earlier the rarefaction and adaptation of the smallest blood vessels before blood hypoperfusion becomes detectable and can be diagnosed. Our findings can serve as targets for interventions aimed at preventing or delaying clinical symptoms. In this context, VAI can detect microvascular responses likely before changes in brain function and lesions become evident. For the future, stimulating revascularization through angiogenesis, combined with blood-brain barrier genesis in the newly formed vessels, seems to be an ideal route to repair or prevent degradation of brain tissue vascularization.^39^ Imaging of the vessel architecture then may provide an early biomarker for selecting patients eligible for future cerebral microvascular therapies and can be utilized in clinical trials to monitor the effects of these interventions.^40^

## Study considerations

Some aspects need to be considered when interpreting the results of our study. First, due to the cross-sectional design of the study, we cannot draw firm conclusions on the timing nor causality of the microvascular alterations in cSVD. Second, using VAI, it is only possible to measure perfused microvessels. Consequently, we cannot infer on the true vessel density also including capillaries that are not perfused in resting state, but that can be recruited in case of increased metabolic demand. Third, VAI is the only *in vivo* MR technique that enables the characterization of microvascular density and radius. Though it has been validated by computer simulations, phantoms, and brain tumour biopsies^11,20,22,41^, the theoretical assumptions underlying VAI harbor certain limitations. For example, simplifications in the model may lead to overestimations of vessel radius.^9^ As such, VAI parameters may not fully reflect the true microvascular structure, especially in pathological contexts like cSVD. Whether the theoretical model is valid in the presence of cSVD-related alterations remains to be demonstrated. For instance, microbleeds or other structural abnormalities may influence the MR signal and thereby affect the accuracy of vessel architecture measurements.^42^

Another consideration in our study concerns the normalization of rCBV values, which directly affects the calculation of the VSI values. This normalization was necessary due to technical limitations in accurately quantifying the arterial input function.^43^ However, it introduces the assumption that average rCBV in NAWM is comparable across groups, an assumption that may not hold in cSVD, where microvascular abnormalities may be (locally) present even in NAWM. To assess the impact of this step, we performed a sensitivity analysis without rCBV scaling. This analysis resulted in rCBV values that did not differ between patients and controls, but the rCBV values were implausibly high and had high variability. Importantly, our key finding – higher VSI in cortical and deep gray matter in both aging and cSVD – remained qualitatively similar (Table S4). However, VSI in NAWM was no longer significantly associated with age or group, and the previously observed association between VSI and sex also disappeared (Table S4). These findings should therefore be interpreted with caution.

Our study also has several strengths. We simultaneously assessed microvessel density, size, and dominating type in a relatively large sample size composed of patients with cSVD and age- and sex-matched controls. Moreover, we used a dynamic vessel size imaging sequence, which has a shorter acquisition time than steady-state vessel size imaging and has the advantage of having temporarily higher gadolinium concentrations and a wider range of relaxation changes. Although the complex post-processing of the GE and SE perfusion images is not automatically available on a clinical scanner, dynamic vessel size imaging (i.e., VAI) could be implemented in clinical practice in the future relatively easily due to its short acquisition time.

## Conclusion

We have demonstrated that cSVD is associated with a lower vessel density, larger vessel size, and a shift from capillaries to an arteriole vessel type. Besides confirming the role of microvascular rarefaction in cSVD, our results suggest that an inhomogeneous capillary bed with a large number of dilated and a few constricted capillaries also features cSVD. Moreover, the microvessel architecture is similarly affected by ageing. VAI might be a promising MRI technique for the detection of early microvascular changes in cSVD, prior to hypoperfusion. Future studies should investigate causality by evaluating longitudinal changes in microvessel architecture, microvessel architecture alterations in relation to disease severity (e.g. the extent of WMH and the severity of cognitive impairment), and whether microvessel rarefaction can be halted by therapeutic strategies.

## Supplemental Material

sj-pdf-1-jcb-10.1177_0271678X251358968 - Supplemental material for Vessel architecture imaging reveals microvascular rarefaction and capillary-to-arteriole shift in cerebral small vessel disease Supplemental material, sj-pdf-1-jcb-10.1177_0271678X251358968 for Vessel architecture imaging reveals microvascular rarefaction and capillary-to-arteriole shift in cerebral small vessel disease by Paulien HM Voorter, Maud van Dinther, Gerhard S Drenthen, Elles P Elschot, Julie Staals, Robert J van Oostenbrugge, Jacobus FA Jansen, Walter H Backes and on behalf of the CRUCIAL consortium in Journal of Cerebral Blood Flow & Metabolism

## References

[1] Pantoni L. Cerebral small vessel disease: from pathogenesis and clinical characteristics to therapeutic challenges. Lancet Neurol 2010; 9: 689–701. 20610345 10.1016/S1474-4422(10)70104-6

[2] Makin SD Turpin S Dennis MS , et al. Cognitive impairment after lacunar stroke: systematic review and meta-analysis of incidence, prevalence and comparison with other stroke subtypes. J Neurol Neurosurg Psychiatry 2013; 84: 893–900. 23457225 10.1136/jnnp-2012-303645 PMC3717603

[3] Joutel A Monet-Leprêtre M Gosele C , et al. Cerebrovascular dysfunction and microcirculation rarefaction precede white matter lesions in a mouse genetic model of cerebral ischemic small vessel disease. J Clin Invest 2010; 120: 433–445. 20071773 10.1172/JCI39733 PMC2810078

[4] Moody DM Thore CR Anstrom JA , et al. Quantification of afferent vessels shows reduced brain vascular density in subjects with leukoaraiosis. Radiology 2004; 233: 883–890. 15564412 10.1148/radiol.2333020981

[5] Hase Y Ding R Harrison G , et al. White matter capillaries in vascular and neurodegenerative dementias. Acta Neuropathol Commun 2019; 7: 16– 12. 10.1186/s40478-019-0666-x PMC6366070 30732655

[6] van Dinther M Voorter PH Jansen JF , et al. Assessment of microvascular rarefaction in human brain disorders using physiological magnetic resonance imaging. J Cereb Blood Flow Metab 2022; 42: 718– 737. 35078344 10.1177/0271678X221076557 PMC9014687

[7] Greene AS Tonellato PJ Zhang Z , et al. Effect of microvascular rarefaction on tissue oxygen delivery in hypertension. Am J Physiol 1992; 262: H1486–H1493. 1590452 10.1152/ajpheart.1992.262.5.H1486

[8] Troprès I Grimault S Vaeth A , et al. Vessel size imaging. Magn Reson Med 2001; 45: 397–408. 11241696 10.1002/1522-2594(200103)45:3<397::aid-mrm1052>3.0.co;2-3

[9] Kiselev VG Strecker R Ziyeh S , et al. Vessel size imaging in humans. Magn Reson Med 2005; 53: 553–563. 15723391 10.1002/mrm.20383

[10] Boxerman JL Hamberg LM Rosen BR , et al. MR contrast due to intravascular magnetic susceptibility perturbations. Magn Reson Med 1995; 34: 555–566. 8524024 10.1002/mrm.1910340412

[11] Weisskoff R Zuo CS Boxerman JL , et al. Microscopic susceptibility variation and transverse relaxation: theory and experiment. Magn Reson Med 1994; 31: 601–610. 8057812 10.1002/mrm.1910310605

[12] Emblem KE Mouridsen K Bjornerud A , et al. Vessel architectural imaging identifies cancer patient responders to anti-angiogenic therapy. Nat Med 2013; 19: 1178–1183. 23955713 10.1038/nm.3289 PMC3769525

[13] Gorelick PB Scuteri A Black SE , et al. Vascular contributions to cognitive impairment and dementia: a statement for healthcare professionals from the American Heart Association/American Stroke Association. Stroke 2011; 42: 2672–2713. 21778438 10.1161/STR.0b013e3182299496 PMC3778669

[14] Duering M Biessels GJ Brodtmann A , et al. Neuroimaging standards for research into small vessel disease—advances since 2013. Lancet Neurol 2023; 22: 602–618. 37236211 10.1016/S1474-4422(23)00131-X

[15] van Dinther M Bennett J Thornton GD , et al. Evaluation of miCRovascular rarefaction in vascUlar cognitive impairment and heArt faiLure (CRUCIAL): study protocol for an observational study. Cerebrovasc Dis Extra 2023; 13: 18–32. 36646051 10.1159/000529067 PMC9939919

[16] Shiroishi MS Castellazzi G Boxerman JL , et al. Principles of T2*‐weighted dynamic susceptibility contrast MRI technique in brain tumor imaging. J Magn Reson Imaging 2015; 41: 296–313. 24817252 10.1002/jmri.24648

[17] Cerri S Puonti O Meier DS , et al. A contrast-adaptive method for simultaneous whole-brain and lesion segmentation in multiple sclerosis. Neuroimage 2021; 225: 117471. 33099007 10.1016/j.neuroimage.2020.117471 PMC7856304

[18] Jenkinson M Bannister P Brady M , et al. Improved optimization for the robust and accurate linear registration and motion correction of brain images. Neuroimage 2002; 17: 825–841. 12377157 10.1016/s1053-8119(02)91132-8

[19] Andersson JL Skare S Ashburner J. How to correct susceptibility distortions in spin-echo echo-planar images: application to diffusion tensor imaging. Neuroimage 2003; 20: 870–888. 14568458 10.1016/S1053-8119(03)00336-7

[20] Troprès I Pannetier N Grand S , et al. Imaging the microvessel caliber and density: principles and applications of microvascular MRI. Magn Reson Med 2015; 73: 325–341. 25168292 10.1002/mrm.25396

[21] Paschoal AM Leoni RF Dos Santos AC , et al. Intravoxel incoherent motion MRI in neurological and cerebrovascular diseases. Neuroimage Clin 2018; 20: 705–714. 30221622 10.1016/j.nicl.2018.08.030 PMC6141267

[22] Kellner E Breyer T Gall P , et al. MR evaluation of vessel size imaging of human gliomas: Validation by histopathology. J Magn Reson Imaging 2015; 42: 1117–1125. 25683112 10.1002/jmri.24864

[23] Elschot EP Backes WH van den Kerkhof M , et al. Cerebral microvascular perfusion assessed in elderly adults by Spin-Echo dynamic susceptibility contrast MRI at 7 tesla. Tomography 2024; 10: 181–192. 38250960 10.3390/tomography10010014 PMC10819808

[24] Boxerman JL Rosen BR Weisskoff RM. Signal‐to‐noise analysis of cerebral blood volume maps from dynamic NMR imaging studies. J Magn Reson Imaging 1997; 7: 528–537. 9170038 10.1002/jmri.1880070313

[25] Wu O Østergaard L Weisskoff RM , et al. Tracer arrival timing‐insensitive technique for estimating flow in MR perfusion‐weighted imaging using singular value decomposition with a block‐circulant deconvolution matrix. Magn Reson Med 2003; 50: 164–174. 12815691 10.1002/mrm.10522

[26] Zhang K Yun SD Triphan SMF , et al. Vessel architecture imaging using multiband gradient-echo/spin-echo EPI. PLoS One 2019; 14: e0220939. 31398234 10.1371/journal.pone.0220939 PMC6688807

[27] Xu C Kiselev VG Möller HE , et al. Dynamic hysteresis between gradient echo and spin echo attenuations in dynamic susceptibility contrast imaging. Magn Reson Med 2013; 69: 981–991. 22611004 10.1002/mrm.24326

[28] Hohmann A Zhang K Jende JME , et al. Vascular architecture mapping reveals sex-specific changes in cerebral microvasculature with aging. Imaging Neuroscience 2024; 2: 1–15. 10.1162/imag_a_00066 PMC12224447 40800419

[29] Shi Y Wardlaw JM. Update on cerebral small vessel disease: a dynamic whole-brain disease. Stroke Vasc Neurol 2016; 1: 83–92. 28959468 10.1136/svn-2016-000035 PMC5435198

[30] Shi Y Thrippleton MJ Makin SD , et al. Cerebral blood flow in small vessel disease: a systematic review and meta-analysis. J Cereb Blood Flow Metab 2016; 36: 1653–1667. 27496552 10.1177/0271678X16662891 PMC5076792

[31] Foda A Kellner E Gunawardana A , et al. Differentiation of cerebral neoplasms with vessel size imaging (VSI). Clin Neuroradiol 2022; 32: 239–248. 34940899 10.1007/s00062-021-01129-8 PMC8894153

[32] Choi H-I Ryu C-W Kim S , et al. Changes in microvascular morphology in subcortical vascular dementia: a study of vessel size magnetic resonance imaging. Front Neurol 2020; 11: 545450. 33192974 10.3389/fneur.2020.545450 PMC7658467

[33] Brown WR Moody DM Thore CR , et al. Vascular dementia in leukoaraiosis may be a consequence of capillary loss not only in the lesions, but in normal-appearing white matter and cortex as well. J Neurol Sci 2007; 257: 62–66. 17320909 10.1016/j.jns.2007.01.015 PMC1989116

[34] Østergaard L Engedal TS Moreton F , et al. Cerebral small vessel disease: capillary pathways to stroke and cognitive decline. J Cereb Blood Flow Metab 2016; 36: 302–325. 26661176 10.1177/0271678X15606723 PMC4759673

[35] Bell MA Ball MJ. Morphometric comparison of hippocampal microvasculature in ageing and demented people: diameters and densities. Acta Neuropathol 1981; 53: 299–318. 7223373 10.1007/BF00690372

[36] Hunziker O Abdel'Al S Schulz U. The aging human cerebral cortex: a stereological characterization of changes in the capillary net. J Gerontol 1979; 34: 345–350. 429767 10.1093/geronj/34.3.345

[37] Berthiaume A-A Schmid F Stamenkovic S , et al. Pericyte remodeling is deficient in the aged brain and contributes to impaired capillary flow and structure. Nat Commun 2022; 13: 5912. 36207315 10.1038/s41467-022-33464-w PMC9547063

[38] Jiménez-Sánchez L Hamilton OKL Clancy U , et al. Sex differences in cerebral small vessel disease: a systematic review and Meta-Analysis. Front Neurol 2021; 12: 756887. 34777227 10.3389/fneur.2021.756887 PMC8581736

[39] Yang Y Torbey MT. Angiogenesis and blood-brain barrier permeability in vascular remodeling after stroke. Curr Neuropharmacol 2020; 18: 1250–1265. 32691713 10.2174/1570159X18666200720173316 PMC7770645

[40] Ip BYM Ko H Lam BYK , et al. Current and future treatments of vascular cognitive impairment. Stroke 2024; 55: 822–839. 38527144 10.1161/STROKEAHA.123.044174

[41] Chakhoyan A Yao J Leu K , et al. Validation of vessel size imaging (VSI) in high-grade human gliomas using magnetic resonance imaging, image-guided biopsies, and quantitative immunohistochemistry. Sci Rep 2019; 9: 2846–2848. 30808879 10.1038/s41598-018-37564-w PMC6391482

[42] Yoo CH Goh J Jahng G-H. Contribution of microbleeds on microvascular magnetic resonance imaging signal. Progress in Medical Physic 2022; 33: 88–100.

[43] Willats L Calamante F. The 39 steps: evading error and deciphering the secrets for accurate dynamic susceptibility contrast MRI. NMR Biomed 2013; 26: 913–931. 22782914 10.1002/nbm.2833

