# Supplementary material for: Vessel architecture imaging reveals microvascular rarefaction and capillary-to-arteriole shift in cerebral small vessel disease
Source: J Cereb Blood Flow Metab. 2025 Jul 31;45(11):2092–103. doi: 10.1177/0271678X251358968 (PMC12316679; doi:10.1177/0271678X251358968)
Supplement: sj-pdf-1-jcb-10.1177_0271678X251358968 - Supplemental material for Vessel architecture imaging reveals microvascular rarefaction and capillary-to-arteriole shift in cerebral small vessel disease [file sj-pdf-1-jcb-10.1177_0271678X251358968.pdf]

Supplemental Material

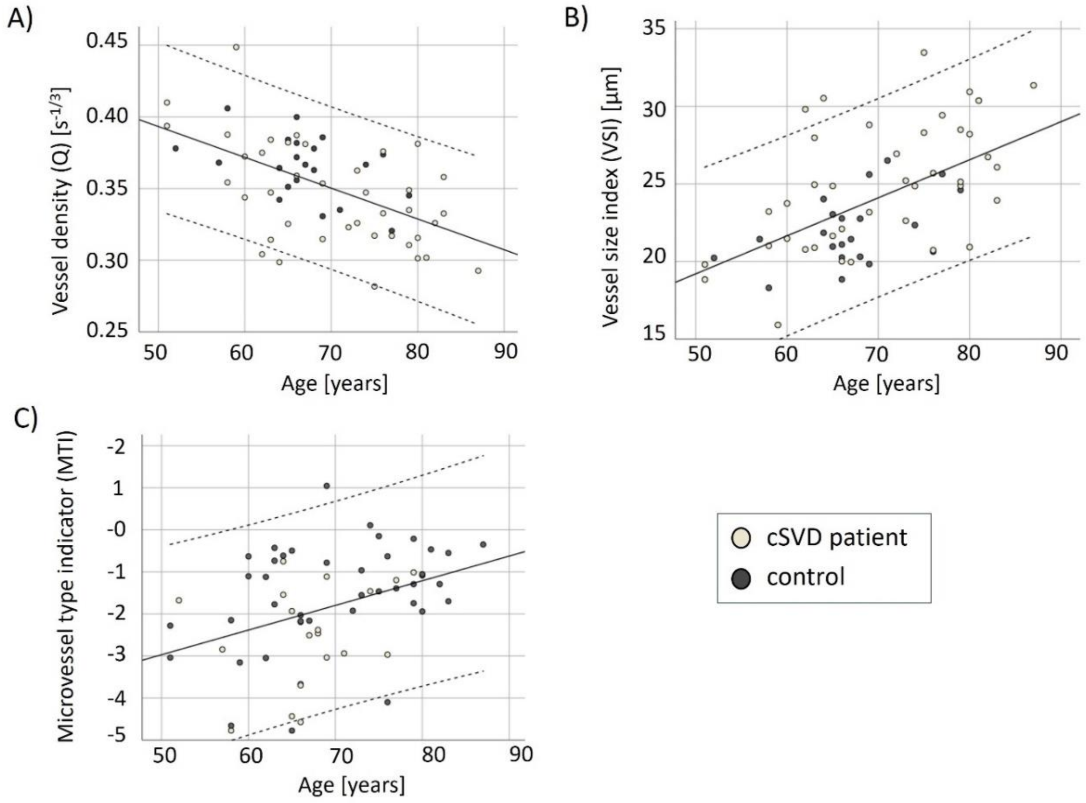

Figure S1. Whole-brain vessel architecture imaging measures in relation to age. A) vessel density decreases with higher age (β=-0.002, *P*<0.001), **B**) vessel size index increases with higher age (β=0.246, *P*<0.001), and **C**) microvessel type indicator increases with higher age (β=0.057, *P*=0.001), indicating a shift from capillaries to arterioles in ageing.

Abbreviations: cSVD indicates cerebral small vessel disease

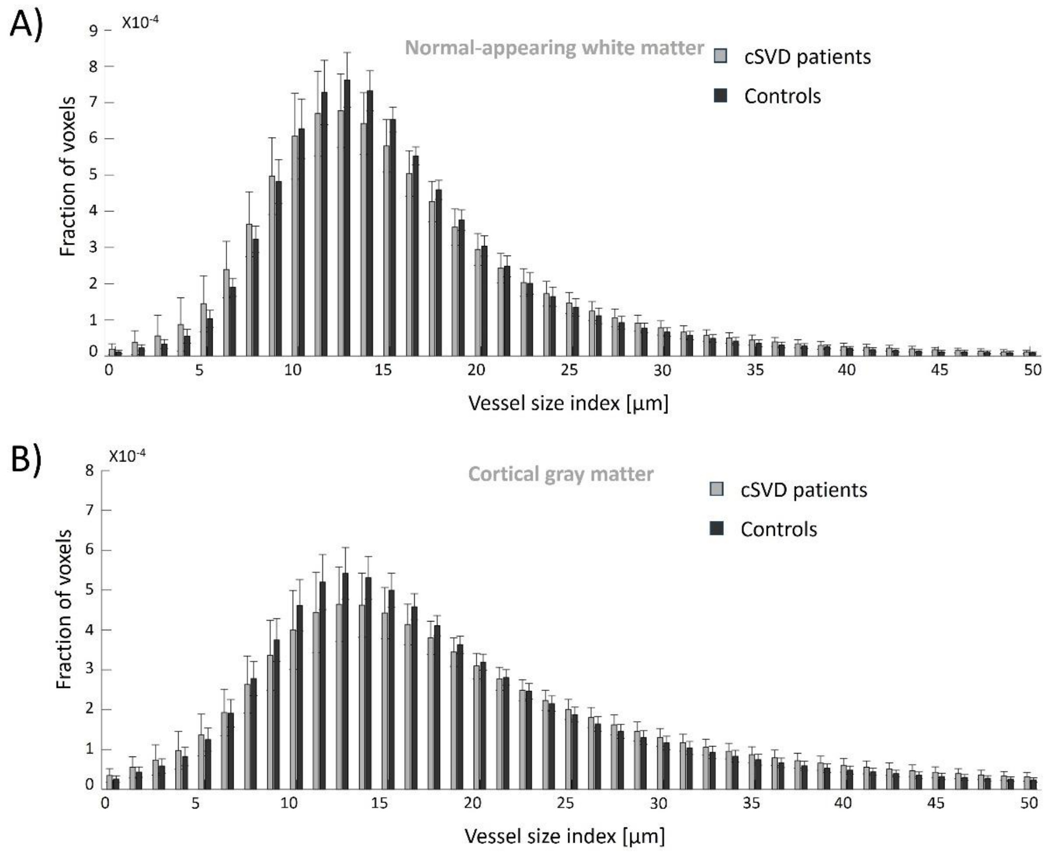

Figure S2. Vessel size index distributions. Vessel size index distributions in **A)** normal-appearing white matter and **B)** cortical gray matter. The distribution of the vessel size index is wider in cSVD patients compared to controls, reflecting a more inhomogeneous microvascular bed in cSVD patients.

Abbreviations: cSVD indicates cerebral small vessel disease

Table S1. Scan parameters of the structural scans.

	T_1-weighted images with two different inversion times	T_2-weighted Fluid-attenuated Inversion Recovery (FLAIR)
Repetition time [ms]	2900	4800
Echo time [ms]	3.8	289
Inversion time(s) [ms]	650 and 2100	1650
Flip angles [degrees]	6 and 6	90
Field of view [mm x mm]	240 x 240	256 x 256
Number of slices	180	180
Voxel size [mm x mm x mm]	1 x 1 x 1	1 x 1 x 1
Acquisition time [min:s]	4:35	6:14

Table S2. Vessel architecture imaging measures in patients with cSVD and controls.

Vessel architecture imaging measure	cSVD patients (N = 40)	Controls (N = 21)	*P*-value
Vessel density index (Q) **[$s^{-1/3}$]**			
WMH*	0.29 ± 0.04		
NAWM	0.39 ± 0.03	0.40 ± 0.01	0.164
DGM	0.37 ± 0.05	0.40 ± 0.04	**0.025**
CGM	0.39 ± 0.04	0.42 ± 0.02	**0.011**
Vessel size index (VSI) **[µm]**			
WMH*	29.0 ± 9.7		
NAWM	17.7 ± 2.3	16.3 ± 0.9	**0.016**
DGM	25.7 ± 6.9	21.2 ± 3.4	**0.007**
CGM	22.7 ± 3.6	19.9 ± 1.5	**0.001**
Microvessel type indicator (MTI)			
WMH*	-1.1 ± 1.1		
NAWM	-0.9 ± 0.7	-1.6 ± 0.8	**0.004**
DGM	-3.8 ± 2.8	-4.1 ± 2.4	0.647
CGM	-2.2 ± 1.7	-3.6 ± 1.9	**0.005**
**CBF_{GE} [ml/min/100g]**			
NAWM	64 ± 14	68 ± 10	0.237
DGM	87 ± 19	94 ± 16	0.197
CGM	90 ± 20	97 ± 15	0.203
**CBF_{SE} [ml/min/100g]**			
NAWM	12.4 ± 2.5	12.8 ± 2.0	0.496
DGM	16.1 ± 3.4	17.3 ± 3.4	0.191
CGM	15.9 ± 3.4	17.1 ± 3.0	0.158

* Vessel architecture imaging measures in WMH were only acquired in cSVD patients, as only patients had substantial WMH volume.

Abbreviations: cSVD indicates cerebral small vessel disease; CBF_{SE}, cerebral blood flow spin echo; CBF_{GE}, cerebral blood flow gradient echo; WMH, white matter hyperintensity; NAWM, normal-appearing white matter; DGM, deep gray matter; CGM, cortical gray matter.

Table S3. Linear regression analyses results for vessel architecture measures when group (0=control, 1=cSVD), age (in years), sex (0=female, 1=male), and cardiovascular risk factors are included as independent variables in the model.

	Regression coëfficient of group		Regression coefficient of cardiovascular risk factor	
	β (95% CI)	*P*-value	β (95% CI)	*P*-value
Additional adjustment for hypertension				
Q				
NAWM	-0.010 (-0.024 – 0.003)	0.136	0.006 (-0.008 – 0.021)	0.388
DGM	-0.029 (-0.052 - -0.006)	**0.014**	0.019 (-0.006 – 0.044)	0.139
CGM	-0.020 (-0.034 - -0.005)	**0.008**	0.001 (-0.014 – 0.016)	0.910
VSI				
NAWM	1.209 (0.229 – 2.189)	**0.017**	-0.344 (-1.401 – 0.712)	0.516
DGM	4.380 (1.352 – 7.408)	**0.005**	-2.044 (-5.307 – 1.219)	0.215
CGM	2.345 (0.975 – 3.715)	**0.001**	-0.031 (-1.508 – 1.445)	0.966
MTI				
NAWM	0.523 (0.137 – 0.908)	**0.009**	0.260 (-0.155 – 0.676)	0.214
DGM	0.079 (-1.140 – 1.298)	0.897	-0.220 (-1.534 – 1.094)	0.738
CGM	1.167 (0.306 – 2.029)	**0.009**	0.470 (-0.459 – 1.398)	0.315
Additional adjustment for diabetes mellitus				
Q				
NAWM	-0.009 (-0.022 – 0.004)	0.446	0.007 (-0.011 – 0.025)	0.446
DGM	-0.026 (-0.049 - -0.003)	**0.029**	-0.007 (-0.038 – 0.024)	0.662
CGM	-0.019 (-0.033 - -0.005)	**0.008**	0.002 (-0.016 – 0.021)	0.797
VSI				
NAWM	1.012 (0.015 – 2.009)	**0.047**	-0.320 (-1.658 – 1.018)	0.634
DGM	3.992 (0.986 – 6.998)	**0.010**	-0.125 (-4.160 – 3.909)	0.951
CGM	2.336 (0.995 – 3.678)	**<0.001**	-0.179 (-1.979 – 1.621)	0.843
MTI				
NAWM	0.567 (0.190 – 0.943)	**0.004**	-0.168 (-0.421 – 0.084)	0.187
DGM	0.028 (-1.160 – 1.216)	0.962	-0.313 (-1.110 – 0.484)	0.435
CGM	1.236 (0.425 – 2.048)	**0.003**	-0.647 (-1.191 - -0.103)	**0.021**
Additional adjustment for BMI				
Q				
NAWM	-0.013 (-0.025 – 0.000)	0.050	0.002 (0.001 – 0.004)	**0.003**
DGM	-0.029 (-0.052 - -0.006)	**0.015**	0.002 (-0.001 – 0.005)	0.131
CGM	-0.023 (-0.036 - -0.010)	**<0.001**	0.002 (0.001 – 0.004)	**0.004**
VSI				
NAWM	1.332 (0.393 – 2.271)	**0.006**	-0.131 (-0.249 - -0.013)	**0.030**
DGM	4.361 (1.353 – 7.368)	**0.005**	-0.255 (-0.633 – 0.123)	0.182
CGM	2.600 (1.293 – 3.908)	**<0.001**	-0.182 (-0.346 - -0.018)	**0.031**
MTI				
NAWM	0.606 (0.221 – 0.992)	**0.003**	-0.024 (-0.072 – 0.024)	0.325
DGM	0.157 (-1.045 – 1.358)	0.795	-0.083 (-0.234 – 0.068)	0.277
CGM	1.410 (0.576 – 2.245)	**0.001**	-0.107 (-0.212 - -0.003)	**0.045**
Additional adjustment for hypercholesterolemia				
Q				
NAWM	-0.012 (-0.027 – 0.002)	0.213	0.010 (-0.006 – 0.027)	0.213

DGM	-0.027 (-0.052 - -0.002)	**0.032**	0.005 (-0.024 – 0.033)	0.741
CGM	-0.021 (-0.036 - -0.006)	**0.008**	0.004 (-0.014 – 0.021)	0.670
VSI				
NAWM	1.191 (0.154 – 2.228)	**0.025**	-0.146 (-1.339 – 1.047)	0.807
DGM	3.812 (0.576 – 7.047)	**0.022**	0.564 (-3.159 – 4.287)	0.763
CGM	2.352 (0.907 – 3.798)	**0.002**	-0.041 (-1.705 – 1.622)	0.960
MTI				
NAWM	0.516 (0.106 – 0.926)	**0.015**	0.174 (-0.298 – 0.646)	0.463
DGM	-0.200 (-1.476 – 1.075)	0.754	0.736 (-0.732 – 2.203)	0.320
CGM	1.217 (0.301 – 2.134)	**0.010**	0.120 (-0.935 – 1.174)	0.821
Additional adjustment for smoking				
Q				
NAWM	-0.009 (-0.023 – 0.004)	0.181	0.000 (-0.018 – 0.018)	0.986
DGM	-0.026 (-0.048 - -0.003)	**0.030**	-0.015 (-0.045 – 0.016)	0.339
CGM	-0.019 (-0.033 - -0.005)	**0.008**	-0.003 (-0.021 – 0.016)	0.771
VSI				
NAWM	1.139 (0.177 – 2.101)	**0.021**	0.247 (-1.029 – 1.524)	0.699
DGM	3.938 (0.990 – 6.887)	**0.010**	2.912 (-0.999 – 6.822)	0.141
CGM	2.336 (0.994 – 3.677)	**<0.001**	0.176 (-1.603 – 1.956)	0.843
MTI				
NAWM	0.572 (0.190 – 0.955)	**0.004**	-0.031 (-0.538 – 0.477)	0.904
DGM	0.026 (-1.163 – 1.215)	0.965	0.602 (-0.974 – 2.179)	0.965
CGM	1.255 (0.403 – 2.106)	**0.005**	0.079 (-1.050 – 1.208)	0.889
Additional adjustment for alcohol				
Q				
NAWM	-0.009 (-0.023 – 0.004)	0.177	0.004 (-0.033 – 0.040)	0.838
DGM	-0.026 (-0.049 - -0.002)	**0.031**	-0.002 (-0.064 – 0.060)	0.943
CGM	-0.020 (-0.034 - -0.006)	**0.007**	0.007 (-0.030 – 0.045)	0.708
VSI				
NAWM	1.171 (0.205 – 2.137)	**0.018**	-0.699 (-3.283 – 1.885)	0.590
DGM	3.967 (0.945 – 6.988)	**0.011**	0.709 (-0.734 – 8.793)	0.861
CGM	2.362 (1.015 – 3.710)	**<0.001**	-0.599 (-4.204 – 3.006)	0.741
MTI				
NAWM	0.569 (0.184 – 0.953)	**0.004**	0.078 (-0.950 – 1.107)	0.879
DGM	-0.004 (-1.201 – 1.192)	0.994	1.084 (-2.116 – 4.285)	0.500
CGM	1.226 (0.374 – 2.079)	**0.006**	0.768 (-1.512 – 3.049)	0.503

Abbreviations: cSVD indicates cerebral small vessel disease; CI, confidence interval; Q, microvessel density; VSI, vessel size index; MTI, microvessel type indicator; NAWM, normal-appearing white matter; DGM, deep gray matter; CGM, cortical gray matter; BMI, body mass index.

Table S4. Regression coefficients of multivariable linear regression analyses for the vessel size index without relative cerebral blood volume normalization.

	Covariable group (0 = control, 1 = patient with cSVD)		Covariable age (in years)		Covariable sex (0 = female, 1 = male)	
	β (95% CI)	*P*-value	β (95% CI)	*P*-value	β (95% CI)	*P*-value
NAWM	2.462 (-1.143 – 6.068)	0.177	0.177 (-0.028 – 0.381)	0.089	-0.633 (-4.304 – 3.037)	0.731
DGM	8.017 (0.615 – 15.419)	**0.034**	0.485 (0.065 – 0.904)	**0.024**	-5.433 (-12.969 – 2.103)	0.154
CGM	4.797 (0.399 – 9.196)	**0.033**	0.417 (0.168 – 0.666)	**0.001**	-2.752 (-7.230 – 1.726)	0.223

Abbreviations: cSVD indicates cerebral small vessel disease; CI, confidence interval; NAWM, normal-appearing white matter; DGM, deep gray matter; CGM, cortical gray matter.
